# It's All in the Mix: Blend-Specific Behavioral Response to a Sexual Pheromone in a Butterfly

**DOI:** 10.3389/fphys.2016.00068

**Published:** 2016-02-29

**Authors:** Helena Larsdotter-Mellström, Kerstin Eriksson, Ilme Liblikas I, Christer Wiklund, Anna K. Borg-Karlson, Sören Nylin, Niklas Janz, Mikael A. Carlsson

**Affiliations:** ^1^Department of Zoology, Stockholm UniversityStockholm, Sweden; ^2^Centre for Evolutionary Biology, The University of Western AustraliaCrawley, WA, Australia; ^3^Department of Organic Chemistry, Institute of Technology, University of TartuTartu, Estonia; ^4^Department of Chemistry, KTH Royal Institute of TechnologyStockholm, Sweden

**Keywords:** butterfly, pieris napi, pheromone, isomer, mixture interactions, calcium imaging, olfaction

## Abstract

Among insects, sexual pheromones are typically mixtures of two to several components, all of which are generally required to elicit a behavioral response. Here we show for the first time that a complete blend of sexual pheromone components is needed to elicit a response also in a butterfly. Males of the Green-veined White, *Pieris napi*, emit an aphrodisiac pheromone, citral, from wing glands. This pheromone is requisite for females to accept mating with a courting male. Citral is a mixture of the two geometric isomers geranial (*E*-isomer) and neral (*Z*-isomer) in an approximate 1:1 ratio. We found that both these compounds are required to elicit acceptance behavior, which indicates synergistic interaction between processing of the isomers. Using functional Ca^2+^ imaging we found that geranial and neral evoke significantly different but overlapping glomerular activity patterns in the antennal lobe, which suggests receptors with different affinity for the two isomers. However, these glomeruli were intermingled with glomeruli responding to, for example, plant-related compounds, i.e., no distinct subpopulation of pheromone-responding glomeruli as in moths and other insects. In addition, these glomeruli showed lower specificity than pheromone-activated glomeruli in moths. We could, however, not detect any mixture interactions among four identified glomeruli, indicating that the synergistic effect may be generated at a higher processing level. Furthermore, correlations between glomerular activity patterns evoked by the single isomers and the blend did not change over time.

## Introduction

Chemical communication is a basis for behavior of most animals, and intra-specific chemical signals—pheromones—have evolved to, for example, induce sexual attraction or to warn conspecifics. Among insects, communication with sexual pheromones has been thoroughly studied particularly in moths (Wyatt, 2014). However, chemical communication in their diurnal lepidopteran relatives, butterflies, has received far less attention. In moths it is usually the female that emits a sexual attractant that may induce upwind search in males over long distance (Wyatt, 2014). In the few species of butterflies studied the sexual pheromones work in a different way. So far, no female-emitted long distance attractants have been identified. Instead, males of the genus *Pieris* emit a pheromone from glands on their wings that act as an aphrodisiac on conspecific females (Andersson et al., 2007; Yildizhan et al., 2009).

The aphrodisiac pheromone in the Green-veined White, *P. napi*, has been identified as citral (Bergström and Lundgren, 1973), which has a strong lemon-like smell to humans. Andersson et al. (2007) showed that female *P. napi* express acceptance behavior when exposed to fresh male wings or wings treated with citral but not to scent-less wings.

**Figure 1:**
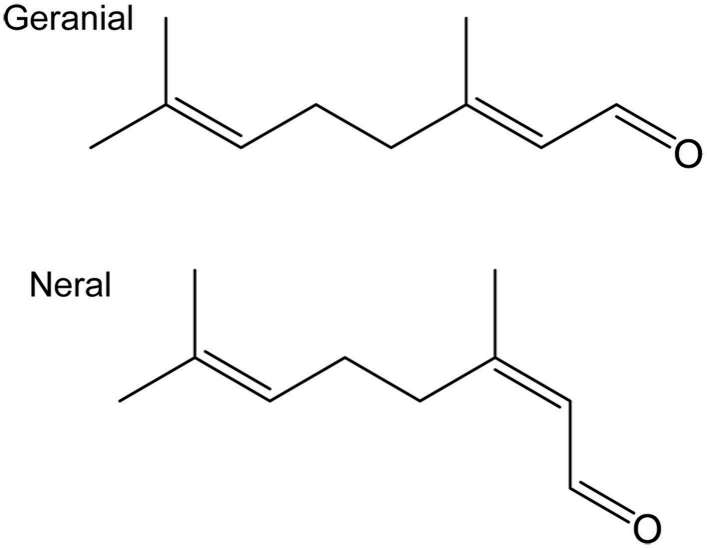
**Chemical structures of the two isomers geranial (*E*-isomer) and neral (*Z*-isomer)**.

Citral is a mixture of two geometric isomers, geranial and neral (Figure 1) in an approximate 1:1 ratio and identified in the wing scent of *P. napi* by Bergström and Lundgren (1973). The adult butterfly can utilize glucose, derived either from larval or adult feeding, to synthesize the pheromone components (Larsdotter-Mellström et al., 2012). In moths and other insects it is, with few exceptions, a blend of two to several components that is required to elicit a behavioral response (Baker, 2011). If a single component of the blend is omitted, there is usually no behavioral response. This is likely because closely related species use a limited array of chemical compounds. The exact composition and ratio of the individual components is crucial as it will prevent interspecific mating attempts. Even sympatric populations of the same species sometimes use different composition of the pheromone, thereby preventing potential matings, which may be an important factor in an on-going speciation process (Carde et al., 1978; Roelofs et al., 1987, 2002; Baker, 2002).

In the present study we aim at investigating female behavioral responses in *P. napi* to citral and the two geometric isomers. In a previous study it was shown that citral elicits an acceptance behavior but the response to the isomers was not tested (Andersson et al., 2007). We hypothesize that only the blend, i.e., citral, would elicit an acceptance response. In addition we report on the physiological responses to the pheromone blend, citral, and their two geometric isomer components, geranial and neral in the primary olfactory center using Ca^2+^ imaging.

## Materials and methods

### Synthesis of geranial and neral

Geranial (*trans*-3,7-dimethyl-2,6-octadien-1-al) and neral (*cis*-3,7-dimethyl-2,6-octadien-1-al) were prepared by separate oxidation of geraniol (natural, = 97%, FG, Aldrich) and nerol (97%, Aldrich), respectively, with four equivalents of manganese (IV)dioxide (MnO_2_) in anhydrous dichloromethane. Reaction products were purified by column chromatography (silica gel Merck 60, 0.040–0.063 mm, gradient elution with increasing amounts of ethyl acetate in petroleum ether). Finally, isomerical purification was done by column chromatography using silica gel impregnated with 12.5 % silver nitrate (by weight). The purity of synthesized compounds was determined on Agilent Technologies 7890A GC System using ß-DexTM 325 fused silica column (Supelco, 30 m, 0.25 mm i.d., 0.25 μm film thickness). Geranial: chemical purity 98.39%, isomeric purity 99.04%. Neral: chemical purity 97.89%, isomeric purity 99.13%.

### Assays of mating behavior with citral and its components

In order to investigate female mate acceptance or refusal behavior when subjected to artificial courtship by scented or unscented male dummies we reared around 150 *P. napi*. The butterflies were all directly developing offspring of >25 females collected in southern Sweden. The larvae were reared in climate cabinets (23°C, 23:1 h light:dark) on the leaves of the natural host plant *Armoracia rusticana*. When an adult butterfly emerged, it was isolated, sex determined and placed in an 8°C refrigerated room. Six males were killed by freezing (−18°C) while the other males were discarded from the experiment. The wings were cut at the base and thereby separated from the body. The natural scent was washed out by two consecutive hour-long immersions in hexane (20°C). Afterwards, the wings were air dried for >24 h. This procedure has been shown to remove naturally occurring fragrance (Andersson et al., 2007).

The females used in the experiment were between 1 and 3 days old. Twenty-four hours before the experiment they were taken out of the cold room and placed in a flight cage (0.7 × 0.7 × 0.5 m, solid plastic walls and a netted opening) to be fully mature at the experiment. In the cage a *Kalanchoe* sp. plant with 20% sugar solution on the flowers, was present for feeding. The air temperature was around 25°C and the room was lit by 400 W HQIL lamps over the cages to simulate daylight between 9 a.m. and 5 p.m. On the morning of the experiment the females were put back in the cold room and each female was then taken out in turn and given around 10 min before each individual trial to warm up in the experiment room and become active.

The male dummies were created from the wings of a frozen male (held together in a resting position by soft forceps) and applied with one of the six treatments ~5 min before the experiment started. The six treatments were scentless wings with either (1) 10 μl of hexane as a control, *n* = 15 (2) 10 μl of citral solution (diluted 1:100 in hexane; ~100 μg), *n* = 21 (3) 5 μl geranial solution (diluted 1:100 in hexane; ~50 μg) *n* = 15, (4) 10 μl geranial solution (diluted 1:100 in hexane; ~100 μg) *n* = 15, (5) 5 μl neral solution (diluted 1:100 in hexane; ~50 μg) n = 15, or (6) 10 μl neral solution (diluted 1:100 in hexane; ~100 μg) *n* = 15. Ninety micrograms of citral has previously been shown by Andersson et al. (2007) to elicit mate acceptance in females. The treatments were applied to the two outer wing surfaces. A total of 96 virgin females were then subjected to 10 artificial courtship bouts by one of the 6 male dummies. Each female was used only for one treatment and then discarded.

The courtship experiment was performed in flight cages as above. At the start of the experiment, one female was released into the cage and when it alighted, the male dummy was presented 10 times in an artificial courtship. The courtship procedure began with the dummy being waved in front of the female to simulate approach and was then brought into physical contact with the wings and antennae of the female (Andersson et al., 2007). This procedure simulates the manner in which male *P. napi* court female conspecifics when alighted under natural conditions (Forsberg and Wiklund, 1989).

Females behave in one of two ways when approached by the dummy: they either reject the dummy (fly away) or accept the courtship (sit still, fold their wings and acquiesce; Forsberg and Wiklund, 1989). For each female we recorded the number of times she accepted the dummy, out of the 10 trials. A new courtship trial was initiated as soon as the female had alighted again or adopted a feeding or resting behavior after accepting or rejecting a courtship bout. Generally 10 trials took ~2 min.

### Functional imaging of olfactory responses

To investigate the sensitivity to citral, geranial, and neral in the butterflies we performed functional Ca^2+^ imaging of the primary olfactory center in the brain, the antennal lobes. The antennal lobes consist of a number of spherical neuropils called glomeruli (in *P. napi* ~65 glomeruli, Larsdotter-Mellström et al., 2016). Each glomerulus receives converging olfactory receptor neurons housing a specific receptor (Couto et al., 2005; Fishilevich and Vosshall, 2005).

Ca^2+^ imaging was performed with a bath applied membrane-permeant fluorescent calcium dye (Calcium Green-1 AM, Molecular Probes, Eugene, OR) dissolved in physiological saline with 20% Pluronic F-127 (Molecular Probes) to a final concentration of 30 μM. This application potentially stains different types of cells in the antennal lobe and generate a compound signal. However, the odor-evoked signal is supposed to originate mainly from input neurons due to the fact that the majority of neurons in a glomerulus are sensory neurons and that they primarily innervate the outer accessible shell (Galizia et al., 1998; Sachse and Galizia, 2003). In addition, with this method we have never observed any spontaneous activity or inhibitory responses, both of which are very common in projection neurons and local interneurons (Hansson and Christensen, 1999). Optophysiological measurements of odor-evoked activity in the AL shows activity in all accessible glomeruli (and thus mainly receptor neurons) responding to a specific volatile. Animal preparation for Ca^2+^ imaging, imaging set up and odor stimulation was identical to previous studies in other butterfly species (Carlsson et al., 2011). In short: animals were placed in a 1000 μl pipette tip with the tip cut open to fit the head. The head protruded at the narrow end was fixed with dental wax. Mouthparts were removed to reduce movements during the experiments. A window was cut between the compound eyes and all tissue covering the brain was removed to uncover the antennal lobes. A drop of Calcium Green-1 AM solution was bath applied to the exposed brain and the preparation was incubated for about 60 min at 8°C. The brain was subsequently rinsed several times with physiological saline to remove excessive dye. Three to seven days old virgin females were used in the experiments.

The imaging set-up consisted of an air-cooled 12-bit slow-scan CCD camera (Olympus U-CMAD3) mounted to an upright microscope (Olympus BX51WI) equipped with a water immersion objective (Olympus, 20x/0.95). Calcium green-1 AM was excited at 475 nm (500 nm SP; xenon arc lamp, Polychrome V, Till Photonics) and fluorescence was detected at 490/515 nm (DCLP/LP). The set-up was controlled by the software Tillvision 4.0 (Till Photonics). Four-fold symmetrical binning resulted in image sizes of 344 × 260 pixels with one pixel corresponding to an area of 1.25 × 1.25 μm.

Ten microliters of citral (Sigma Aldrich, Saint Louis, MO, USA), geranial, or neral diluted in hexane or 10 μl of hexane (control) were applied onto a rectangular piece of filter paper (20 × 5 mm). All compounds were diluted in decadic steps from 1:100-1:10 000 (v/v). In addition, we tested a number of other volatile compounds:: methyl salicylate (Sigma Aldrich), benzyl cyanide (Lancaster, Ward Hill, MA, USA), (−)-linalool (KTH, Stockholm, Sweden), phenyl acetaldehyde (Sigma Aldrich), hexanol (Sigma Aldrich), methyl benzoate (Lancaster), allyl isothiocyanate (Sigma Aldrich), and nonanal (Sigma Aldrich) all at 1:1000 v/v. All filterpapers were left in a fumehood for a couple of minutes to allow the hexane to evaporate. When citral and the isomers were compared the total odor concentration was the same, i.e., citral 1:100 means a mixture of geranial 1:200 and neral 1:200.

Filter papers were inserted into glass Pasteur pipettes and were renewed every day. A humidified and charcoal-filtered continuous air stream (1 l/min) was ventilating the antenna ipsilateral to the recorded antennal lobe through a glass tube (5 mm inside diameter). The glass tube ended ~10 mm from the distal part of the antenna. An empty Pasteur pipette was inserted through a small hole in the glass tube, blowing an air stream of 0.1 l/min. Another air stream (0.1 l/min) was blown through the odor-laden pipette by a computer-triggered puffer device (Syntech, Hilversum, The Netherlands) during 2 s (starting at frame 12) into the continuous stream of air. During stimulation, the air stream was switched from the empty pipette to the odor-laden one in order to minimize the influence of added air volume. Each recording consisted of a sequence of 50 frames obtained at 4 Hz. We used at least 60 s interstimulus periods to reduce adaptation. Each odor was tested 1–3 times depending on the condition of the animal and the stimulus order was randomized.

### Imaging analysis

To analyse the obtained Ca^2+^ imaging data we constructed false-color coded images of relative changes of fluorescence intensity during the peak time of activity (frame 16–21). By visual inspection of activity maps we drew circular regions of interest (20 pixels diameter corresponding to 25 μm, which is roughly 50% of the diameter of an average glomerulus) round all centers of elevated activity. Eight to eleven activity foci were observed at the higher concentrations and these regions were used to construct activity maps. An additional region of interest was drawn in an area with minimal activity, which served to correct for bleaching. In an earlier study in moths it was demonstrated that activity foci correspond to individual glomeruli (Carlsson and Hansson, 2003). The mean pixel value within a region of interest was calculated for each time-point in a sequence and exported to Microsoft Excel. In Excel we first made a temporal median filtering of data over three consecutive frames. Secondly we calculated the relative fluorescence (dF/F) where F was defined as the mean value of frames 3–10. To correct for bleaching we subtracted the values of the control region of interest from the values of activity region of interest for each recording. A response was finally defined as the mean of frames 16–21 (peak of activity). This means that each odor-evoked response is represented by a vector in an 8–11-dimensional space (i.e., responses in the 8–11 activated activity foci or glomeruli). In each animal we calculated the correlation index between the vectors of all odor pairs (Pearson linear test; JMP SAS, Cary, NC, USA). The correlation indices for same and different odor pairs were compared by Kruskal–Wallis test followed by Dunn's multiple comparison's test (Prism 5, GraphPad Software, Inc.). The same test was used to compare responses to pheromone and other compounds and to compare correlation indices between time points. Calculation of differences between dose-response curves for single glomeruli were done using a two-way ANOVA (Prism 5, GraphPad Software, Inc.). To compare glomerular activity patterns evoked by either the single isomers or citral at two time points we used Wilcoxon matched-pairs signed rank test (Prism 5, GraphPad Software, Inc.).

## Results

### Mating behavior with citral and its components

**Figure 2:**
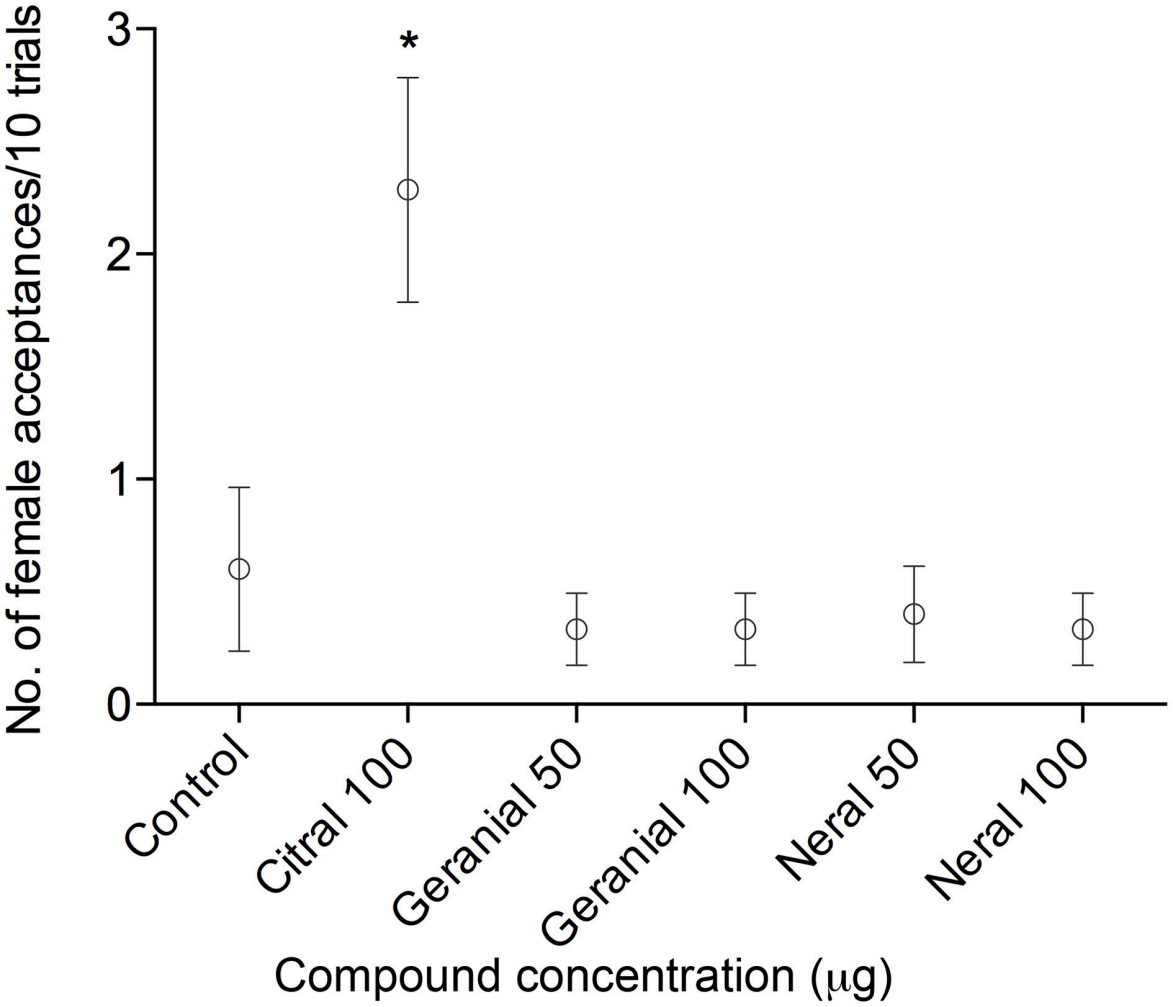
**Female acceptance behavior**. A male dummy consisting of washed wings held together with a forceps was waved in front of an alighted sexually mature female (10 trials/female). The wings were coated with citral, geranial, neral, or a solvent control. The female either accepted mating or flew away. Each point represents the mean of acceptances of 10 trials. Only wings coated with citral elicited a significant acceptance behavior. (Kruskal–Wallis X^2^ = 22.97, df = 5, *p* = 0.0003, Wilcoxon *post hoc* ^*^p < 0.05, *n* = 15–21 in each treatment).

Females behaved in one of two ways when approached by the dummy: they either flew away (rejection) or sat still, folded their wings and acquiesced (acceptance; Forsberg and Wiklund, 1989). Females showed acceptance behaviors (2.30 +∕− 0.50, mean number of acceptances out of 10 presentations for each animal +∕−SEM, *n* = 21, Figure 2) when exposed to the dummy treated with citral. This was significantly different (Kruskal–Wallis X^2^ = 22.97, df = 5, *p* = 0.0003) from all other treatments (control 0.60 +/−0.36 *n* = 15, 50 μg geranial 0.33 +/−0.16 *n* = 15, 100 μg geranial 0.33 +/−0.16 *n* = 15, 50 μg neral 0.40 +/−0.22 *n* = 15, 100 μg neral 0.33 +/−0.16 *n* = 15, *post-hoc* test Wilcoxon pairwise tests with Bonferroni correction *P* < 0.05).

Next we investigated physiological responses to citral, neral and geranial in the primary olfactory center, the antennal lobes, by means of functional Ca^2+^ imaging. We measured changes in [Ca^2+^] in focal activity regions, corresponding to individual olfactory glomeruli (Carlsson and Hansson, 2003).

**Figure 3:**
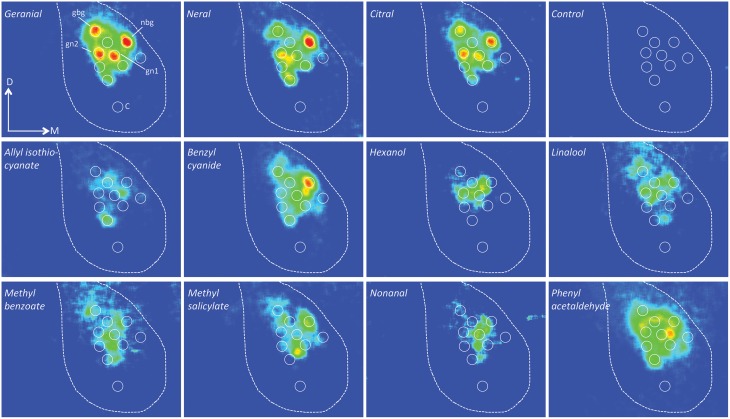
**False color coded images of glomerular activity responses in a female *P. napi***. The upper row show responses to the pheromone isomers, citral, and a control (hexane). The two lower row shows responses to eight different non-pheromonal compounds. The images show the relative change in activity (dF/F) during stimulation. For comparison, all images have identical intensity scales. The border of the antennal lobes are depicted by the dotted line. All activity foci (and control region) used for analysis in this animal are shown as circular regions. The geranial-best (gbg), the neral-best (nbg), gn1 and gn2 glomeruli as well as the control region (C) are marked. D, dorsal; M, medial.

**Figure 4:**
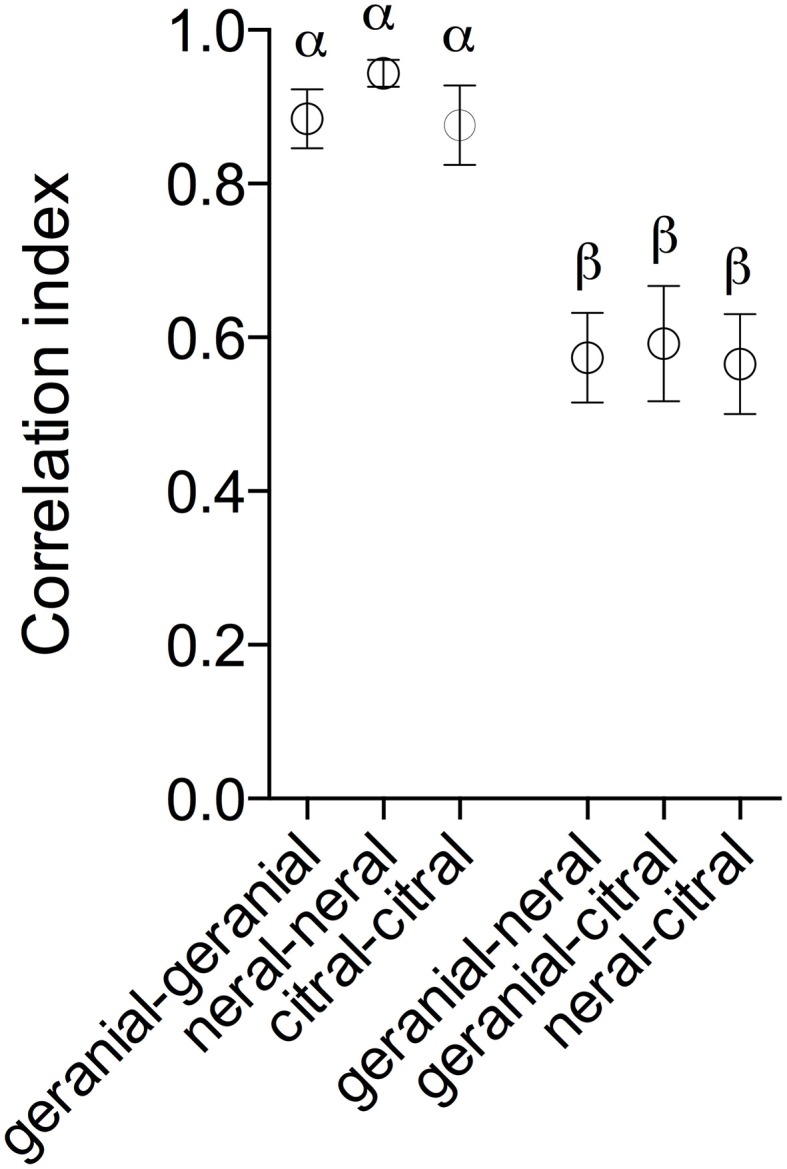
**Mean correlation indices (+∕−SEM) for comparisons of responses evoked by either repeated stimulation with the same odorant (geranial-geranial, neral-neral, and citral-citral) or between odorants (geranial-neral, geranial-citral, and neral-citral)**. Circles capped by different letter differ significantly (*p* < 0.01, *n* = 7; Kruskal–Wallis test followed by Dunn's multiple comparison's test).

All compounds evoked activity in several glomeruli as shown in false color coded images in Figure 3. At the higher concentrations up to 11 glomeruli (8–11) were activated. For comparison we also show images of responses to eight plant-related compounds and other semiochemicals (Figure 3). When activity patterns were compared by correlation analysis (Pearson linear test) we found that neral, geranial, and citral evoked significantly different responses. Correlation indices between repeated activation by the same compound (neral-neral, geranial-geranial or citral-citral; 0.94 +/−0.02, 0.88 +/−0.04, 0.88+∕−0.05; mean +∕−SEM) were significantly higher than correlations between patterns evoked by different compounds (geranial-neral, geranial-citral, neral-citral; 0.57 +/−0.06, 0.59 +/−0.07, 0.56 +/−0.06; *p* < 0.01, *n* = 7; Kruskal–Wallis test followed by Dunn's multiple comparisons test, Figure 4). However, no differences were found between correlations of repeated stimuli or between correlations of different stimuli.

**Figure 5:**
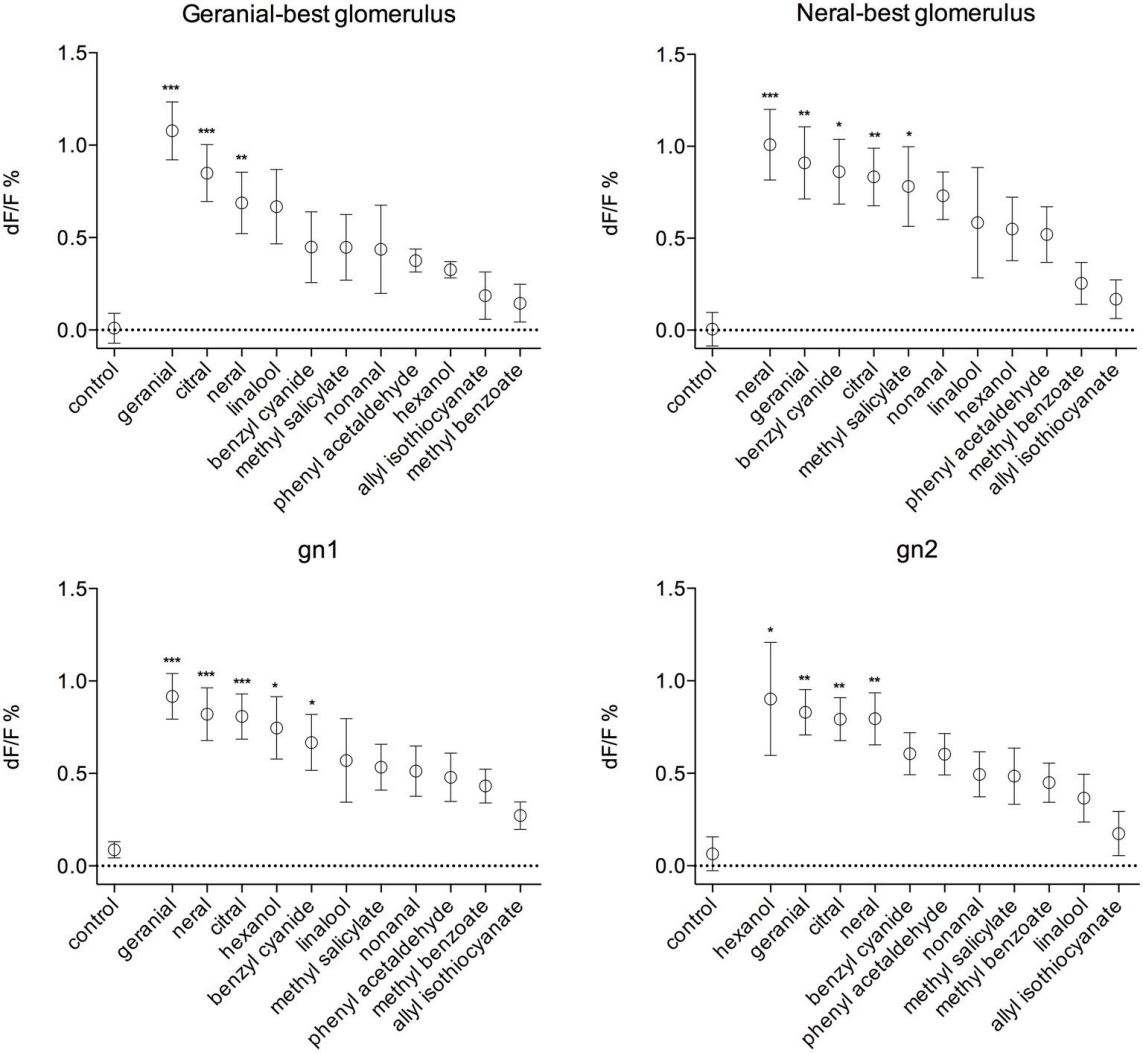
**Mean intensity change (dF/F, %+∕−SEM) for 11 different compounds plus a hexane control in four glomeruli, the geranial-best, the neral-best, gn1, and gn2**. All compounds were used at the concentration 1:1000. In the geranial-best glomerulus only geranial, neral, and citral evoked significantly higher responses than the control. In the neral-best glomerulus neral, geranial, citral, benzyl cyanide, and methyl salicylate evoked higher responses than the control. The gn1 responded to hexanol and benzyl cyanide and gn2 to hexanol in addition to the pheromone and its components. All responses (except the control) are ranked according to the mean values. ^*^p < 0.05, ^**^p < 0.01, ^***^p < 0.001, *n* = 4−10 (Kruskal–Wallis followed by Dunn's multiple comparison's test).

As activity patterns evoked by citral and its components seemingly overlap with patterns evoked by other odorants, we measured responses to a number of other compounds with differing biological relevance in four physiologically identified glomeruli (Figure 5). Two of these glomeruli showed strongest response to neral and geranial, respectively, at the concentration 1:1000. The geranial-best glomerulus (gbg) was located dorsal to the neral-best glomerulus (nbg) in all animals (Figure 3). In the geranial-best glomerulus only geranial (*p* < 0.001), neral (*p* < 0.01), and citral (*p* < 0.001) showed a significantly stronger response than the control (Kruskal–Wallis followed by Dunn's multiple comparison's test). In the neral-best glomerulus responses to geranial (*p* < 0.01), neral (*p* < 0.001), citral (*p* < 0.01), methyl salicylate (*p* < 0.05), and benzyl cyanide (*p* < 0.05) showed significantly stronger responses than the control. The two latter compounds are both anti-aphrodisiac pheromones in *P. napi* and *P. brassicae*, respectively (Andersson et al., 2000, 2003). Two additional glomeruli were identified as they strongly responded to both geranial and neral. Moreover, the positions of these glomeruli were conserved across animals (Figure 3). We call them gn1 and gn2, respectively, where gn1 is the most medially located of the two. Both gn1 and gn2 responded to hexanol (Figure 5). GN1 additionally responded to benzyl cyanide.

**Figure 6:**
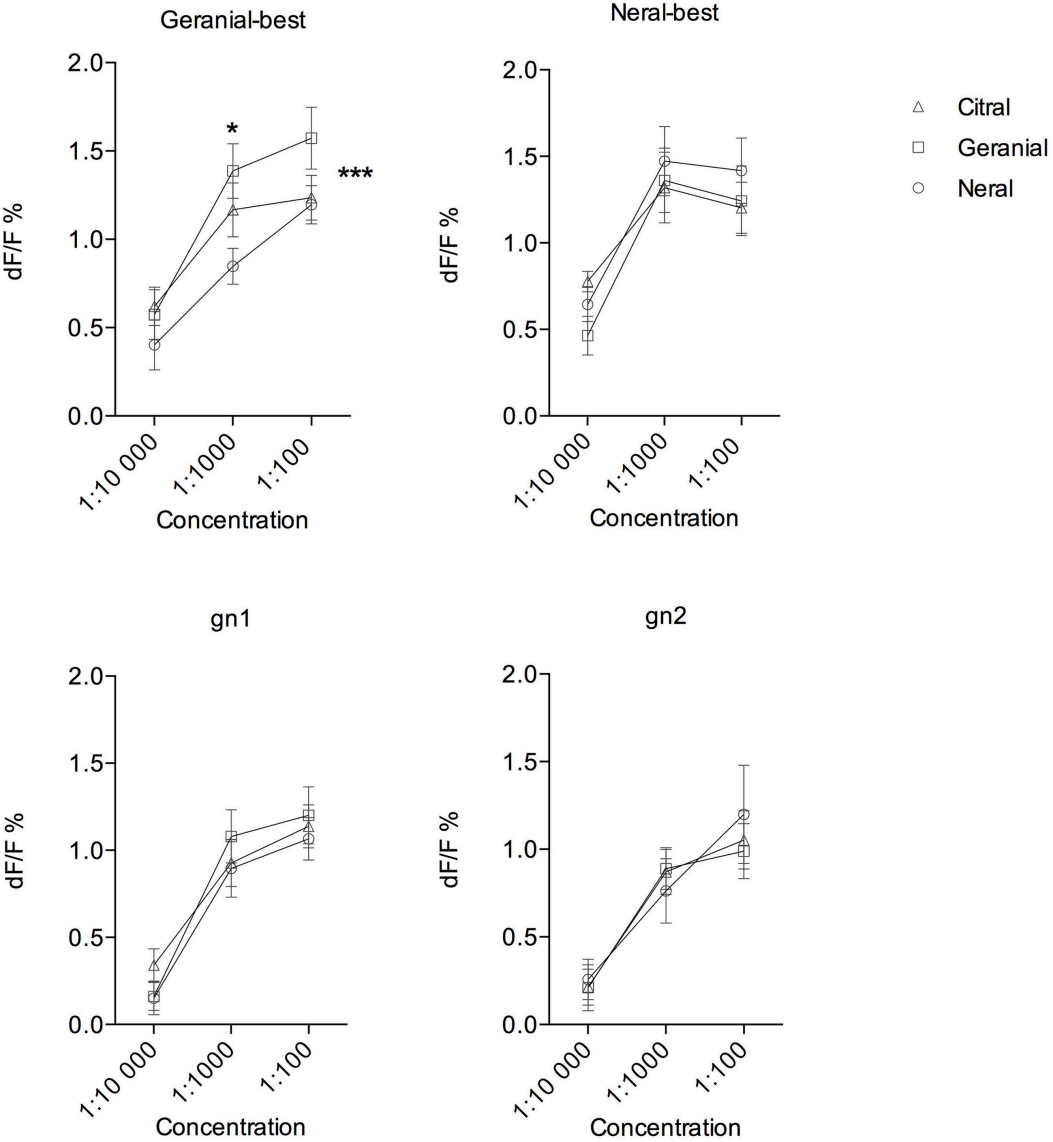
**Dose-response analysis of odor-evoked activity in four glomeruli (geranial-best, neral-best, gn1, and gn2) to geranial, neral, and citral at three concentrations**. In the geranial-best glomerulus responses to the compounds differ significantly (^***^p < 0.0001 *n* = 10; two-way ANOVA). A *post hoc* test showed a difference between geranial and neral at 1:1000 (^*^p < 0.05, Tukey's multiple comparisons test). In the other three glomeruli no difference was detected.

Next we measured the dose-response activity to neral, geranial and citral in the four identified glomeruli (Figure 6). In the geranial-best glomerulus we found a significant difference between odorants (*p* < 0.0001 *n* = 10; two-way ANOVA). A *post hoc* test showed a significant difference between geranial and neral at the concentration 1:1000 (*p* < 0.05, Tukey's multiple comparison's test). However, citral did not differ from either geranial or neral. In the neral-best glomerulus, gn1, and gn2, on the other hand, no difference between odorants was found. For these three glomeruli there was also a significant effect of dose (*p* < 0.0001) but no interaction between dose and odorant.

**Figure 7:**
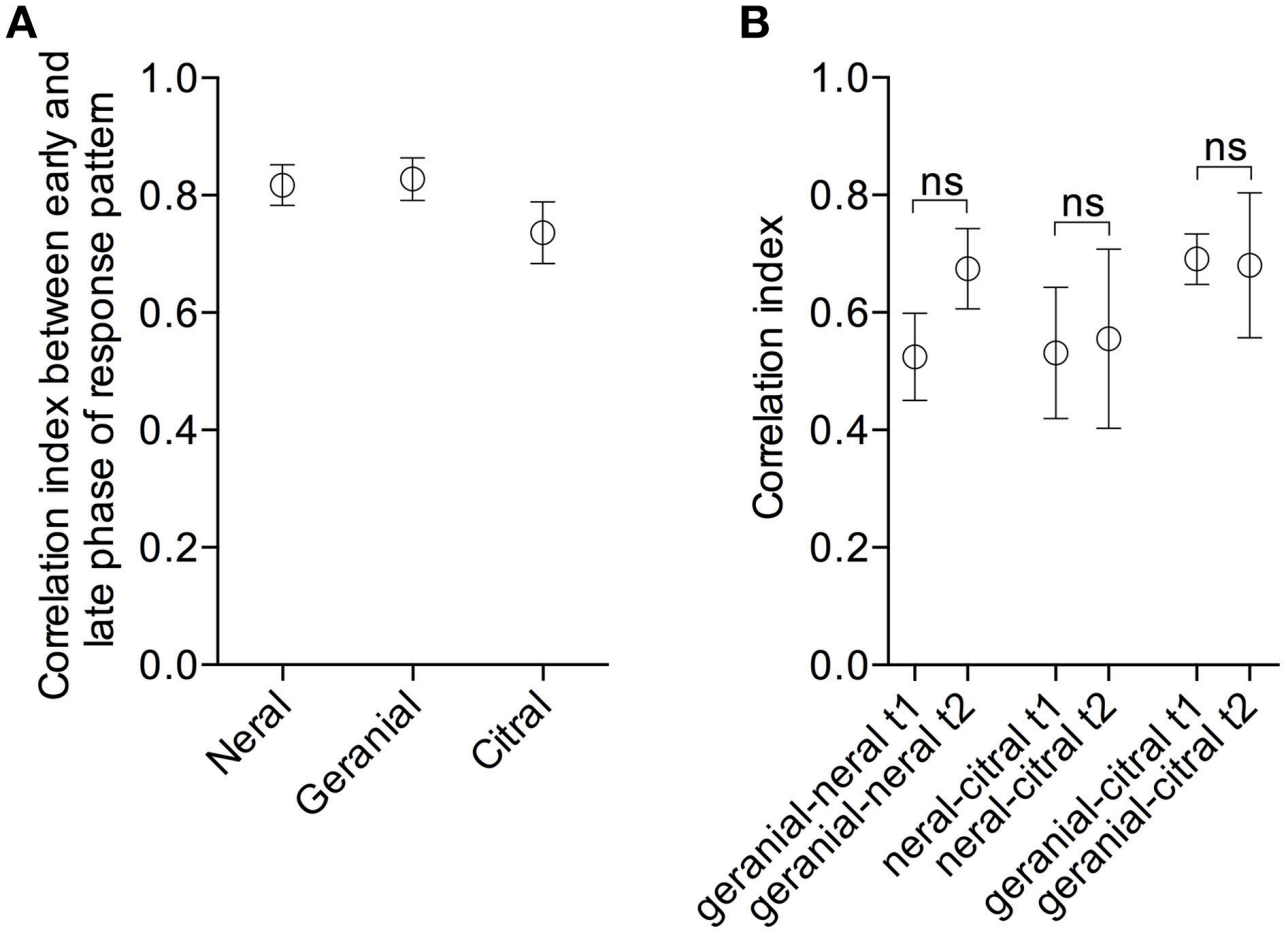
**(A)** Mean correlation indices (+∕−SEM) for comparisons of glomerular activity patterns evoked at the start (frame 15) and at the end (frame 24) of the activity period for neral, geranial and citral, respectively. No significant difference between the compounds (Kruskal-Wallis *X*^2^ = 2.555, df = 2, *p* = 0.2788, *n* = 9). **(B)** Mean correlation indices (+∕−SEM) for odorant pairs at t1 and t2 (frame 15 and 25, respectively). Correlation indices were compared at the two time points. No significant differences between time points were found for any of the odorant pairs (Wilcoxon matched-pairs signed rank test).

In a previous study we found that glomerular activity patterns in *Spodoptera littoralis* are temporally dynamic though highly repeatable during the short period of stimulus exposure (Carlsson et al., 2005). As we in this study used mean of intensity around the peak of activity it cannot be ruled out that mixture interactions may occur during this window and that the response to citral changes over time due to lateral processing. Therefore, we calculated the correlations (Pearson linear test) between glomerular activity patterns at the start of [Ca^2+^] increase (frame 15, time point 1, t1) and at the end (frame 24, t2). Correlation of activity patterns (Figure 7) were, however, high for all compounds (0.82 +/−0.03 for neral, 0.83 +/−0.04 for geranial and 0.74 +/−0.05 for citral), i.e., activity patterns are relatively static over time. Most importantly, correlation indices did not significantly differ from each other (Kruskal–Wallis X^2^ = 2.555, df = 2, *p* = 0.2788, *n* = 9). That is, the patterns evoked by citral did not differ more over time than the patterns evoked by either of the two isomers. Finally, we calculated the correlations between patterns evoked by either the single isomers or citral at t1 and t2. For none of the odorant pairs did we find any significant change in correlation from t1 to t2 (geranial-neral *p* = 0.195, neral-citral *p* = 0.945, geranial-citral *p* = 0.250; Wilcoxon matched-pairs signed rank test).

## Discussion

In the present study we first investigated female behavioral responses to the aphrodisiac pheromone citral and its geometric isomer components. We found that females showed acceptance behavior only in the presence of citral but not to the individual components, geranial and neral. The male dummies coated with geranial or neral solutions evoked significantly less female acceptance behavior than the dummy treated with citral solution, regardless of amount. In fact, the dummies treated with geranial or neral (50 or 100 μg) did not evoke behaviors different from that evoked by the control dummies treated with hexane. This indicates that both components of citral are necessary in order to evoke female acceptance behavior and suggests a strong synergy between geranial and neral.

Sexual pheromones in moths are with few exceptions blends of two to several compounds and the complete blend is generally necessary to elicit behavior. For example, the moth *Helicoverpa zea* uses a two-component blend consisting of structurally similar compounds, (Z)-11-hexadecenal and (Z)-9-hexadecenal and both components are required to elicit a male response (Klun et al., 1979, 1980; Sparks et al., 1979). Here we show for the first time that a complete blend of pheromone components is necessary to evoke a response also in butterflies.

Furthermore, Ca^2+^ imaging of odor-evoked AL activity showed that geranial and neral evoked significantly different, though overlapping, activity patterns. This means that different receptors have different affinities for the two compounds, despite their structural similarity. However, the response to citral was generally not stronger (or weaker) than responses to any of the compounds in the four identified glomeruli, implying that synergistic processing may occur at higher integration levels in the brain. The possibility remains that other glomeruli with weaker responses that could not be identified across animals may elicit synergistic responses. In the geranial-best glomerulus the response to geranial was significantly stronger than the response to neral. However, the response to citral did not differ from either of the components. This implies that this receptor (as indirectly measured from the sensory neuron terminals in this glomerulus) has different affinities for geranial and neral. The response to citral was not significantly different from either of the isomers but was generally an intermediate of responses to neral and geranial, which would be expected as citral consists of a 1:1 mixture of the isomers. In the case of the neral-best glomerulus we did not find a difference between responses to any of the compounds. This may imply that the receptor neurons innervating this glomerulus have the same affinity for geranial and neral. If so it makes sense that the response to citral is equal to the responses to its components, i.e., a mixture of an equipotent dose of geranial and neral (e.g., 5 mg of each as in the citral) would act as a double dose of either geranial or neral (i.e., 2 × 5 mg). Two additional glomeruli were analyzed where we observed strong response to the isomers and citral, though weaker than in the geranial- and neral-best glomeruli. Responses in these glomeruli appeared similar as in the neral-best glomerulus, that is equal response to both the isomers and to citral. Thus, our results indicate that the four receptors are activated in different ways to geranial and neral, either with equal or with different affinity. Consequently, a neural basis exists that may enable behavioral discrimination between the isomers and the mixture.

Citral activates a subpopulation of 8–11 glomeruli to a different degree. These glomeruli intermingle and overlap with glomeruli responding to other compounds. As we used a bath applied Ca^2+^ sensor, which primarily mirrors the input to the glomerulus (Galizia et al., 1998; Sachse and Galizia, 2003) and as each glomerulus is only innervated by neurons housing a specific receptor (Hansson et al., 1992; Couto et al., 2005; Fishilevich and Vosshall, 2005; Lee et al., 2006) we can draw the conclusion that several different receptors can be activated by neral and geranial. Moreover, in an earlier study we showed that male and female glomeruli are not sexually dimorphic (Larsdotter-Mellström et al., 2016). This is a clear contrast to how sexual pheromones are processed in male moths. In moths, a cluster of glomeruli in the male specific macroglomerular complex (MGC) responds to the pheromone and each glomerulus within the MGC responds specifically to one of the components. The MGC is always clearly separated from “ordinary” (plant-odor responding) glomeruli. In addition, studies of pheromone activation of single cells at the antennal level have shown extreme specificity, in that individual cells only respond to a single component even at very high concentrations (Carlsson and Hansson, 2002). Thus, moths and butterflies use different strategies to code for pheromone components. Butterflies use unique and overlapping patterns of activated glomeruli (like food odor coding) whereas moths use a labeled line strategy with specific glomeruli for each pheromone component. However, both ways of coding can be used to distinguish between the components. It is not unlikely that the reception pathways for citral in *P. napi* may have evolved to detect and perceive this compound solely as a plant compound. When males evolved the emission of the aphrodisiac pheromone the female processing system was already present, i.e., perception by across-glomerular coding.

It should be noted that female moths can often autodetect their pheromone despite lack of an MGC and may thus use the food-odor glomeruli instead (Stelinski et al., 2014). Furthermore, many moth species use male-emitted close range pheromones, just as butterflies (Birch et al., 1990; Hillier and Vickers, 2011; Sarto i Monteys et al., 2012). Interestingly, one such moth species, *Paysandisia archon*, is diurnal and has lost the use of a long-range female emitted pheromone (Sarto i Monteys et al., 2012).

The fact that there is no clear separation between pheromone and plant odorant-responding glomeruli is, however, not unique to butterflies. In honeybees, e.g., the glomeruli responding to isoamyl acetate, which is a component of the sting pheromone, are also overlapping with glomeruli responding to floral and other plant-related odorants (Galizia et al., 1999). Isoamyl acetate is in fact also present in some plant species, e.g., banana. Interestingly, citral is also present in several plant species as e.g., lemongrass and orange (Martins et al., 2011). Therefore, citral emitted from plants may potentially interfere with the pheromone communication in the butterflies. The context and interaction with other sensory modalities may, however, play a crucial role. Interestingly, three of the physiologically identified glomeruli also significantly responded to other compounds. In addition to citral and it's components, the neral-best glomerulus responded to methyl salicylate and benzyl cyanide. Both these compounds have been demonstrated to work as anti-aphrodisiac pheromones in *P. napi* and *P. brassicae*, respectively (Andersson et al., 2000, 2003). The other two identified glomeruli, which showed weaker responses to the isomers and citral, also responded to benzyl cyanide (gn1) and hexanol (gn1 and gn2). The degree of specificity in pheromone-responding neurons obviously differ between animal groups. In moths sensory neurons responding to pheromone components are extremely specific and do not normally respond even to closely related compounds (Carlsson and Hansson, 2002; Baker et al., 2004; Liu et al., 2013). However, an exception was recently reported in that a plant volatile could act as an agonist on pheromone sensing neurons in the moth *Agrotis ipsilon* (Rouyar et al., 2015). Even higher order neurons, i.e., after processing in the antennal lobe, frequently respond with high specificity to single pheromone components (Lei and Hansson, 1999). In contrast, the geometric isomers neral and geranial both activate the same glomeruli (and thus receptors) but with different affinities. In addition, at least the neral-best glomerulus (and receptor) could be activated by other compounds.

In our study we could not detect any synergistic responses in the studied glomeruli matching the observed. Hence, the neural basis for the behavioral mixture interaction may be present at a higher integration level. In fact, lack of mixture interactions at the input level is rather a rule (Carlsson and Hansson, 2002; Deisig et al., 2006; Carlsson et al., 2007; Silbering and Galizia, 2007). On the other hand, at the next level, i.e., the projection neurons postsynaptic to the sensory neurons, non-linear responses to mixtures are ubiquitous (Silbering and Galizia, 2007; Deisig et al., 2010). Furthermore, we did not detect any changes in activation patterns over time, i.e., the response at the onset resembles the patterns at the offset. This is in contrast to a previous study in the moth *S. littoralis* showing remarkable changes in glomerular representations and diminished correlation between patterns evoked by different volatiles (Carlsson et al., 2005). However, in the latter study recordings were done specifically from projection neurons. Nonetheless, it cannot be ruled out that other forms of mixture interactions may occur that are not detectable with the method used in this study. For example, a pheromone blend or floral odor mixtures in the moth *Manduca sexta* generate synchronous firing between neurons (Riffell et al., 2009; Martin et al., 2013). It should be emphasized that the technique we have used have obvious limitations regarding both the identity of the recorded neurons and the temporal resolution. However, its clear advantage is to simultaneously and repeatedly show responses in a large number of activated neurons and thus study odor-evoked spatial activity patterns.

Citral has a behavioral significance also in males in that they use it to assess male density, and thus competition, and are able to tailor their ejaculate according to this assessment (Larsdotter-Mellström and Wiklund, 2009). Males respond to citral (and its isomers) in similar way as females, i.e., with activation in a sub population of glomeruli intermingled with glomeruli responding to plant-related compounds (data not shown). Another *P. napi* pheromone, methyl salicylate, which is transferred by males to females during copulation, also functions as an assessment signal for tailoring ejaculates (Larsdotter-Mellström et al., 2016). Thus, citral has dual functions in *P. napi* and it would be very interesting to continue to investigate how citral is processed at higher integration levels in the brain, and how it is connected to the reproductive system in males and females. Sexually dimorphic neural pathways have been demonstrated in the fruitfly, in which the pheromone cVA evokes different behavior in males and females (Datta et al., 2008).

In conclusion, sexual pheromone processing in *P. napi* is similar to moths, in that all components of a pheromone are generally required to elicit a behavioral response. However, female *P. napi* use an across-neuron (and glomerular) coding strategy with several different receptor neurons with different affinities whereas male moths use a labeled line strategy with highly specific receptor neurons and glomeruli. Anyhow, the isomeric components of the pheromone clearly evoked different responses in the antennal lobe, which is a prerequisite for the neural system to generate a synergistic effect. Nevertheless, we could not demonstrate a neural blend interaction at the first level of processing (at least not in the glomeruli we could identify) mirroring the behavioral synergistic effect. Such processing is thus likely generated at a higher level in the olfactory pathway, i.e., down stream of the receptor neurons.

## Author contributions

HL and MC wrote the manuscript. HL, CW, AB, SN, NJ, and MC designed experiments. IL and AB performed the synthesis of the isomers. HL and KE performed the behavioral experiment. MC performed the functional imaging experiment and analyzed data. All authors reviewed the manuscript.

### Conflict of interest statement

The authors declare that the research was conducted in the absence of any commercial or financial relationships that could be construed as a potential conflict of interest.

## References

[B1] Andersson J. Borg-Karlson A. K. Vongvanich N. Wiklund C. (2007). Male sex pheromone release and female mate choice in a butterfly. J. Exp. Biol. 210, 964–970. 10.1242/jeb.02726 17337709

[B2] Andersson J. Borg-Karlson A. K. Wiklund C. (2000). Sexual cooperation and conflict in butterflies: a male-transferred anti-aphrodisiac reduces harassment of recently mated females. Proc. R. Soc. Lond. B Biol. 267, 1271–1275. 10.1098/rspb.2000.1138 10972120 PMC1690675

[B3] Andersson J. Borg-Karlson A. K. Wiklund C. (2003). Antiaphrodisiacs in pierid butterflies: a theme with variation! J. Chem. Ecol. 29, 1489–1499. 10.1023/A:1024277823101 12918930

[B4] Baker T. C. (2002). Mechanism for saltational shifts in pheromone communication systems. Proc. Natl. Acad. Sci. U.S.A. 99, 13368–13370. 10.1073/pnas.222539799 12374851 PMC129676

[B5] Baker T. C. (2011). Insect pheromones: useful lessons for crustacean pheromone programs?, in Chemical Communication in Crustaceans, eds Breithaupt T. Thiel M. (New York, NY: Springer), 531–550.

[B6] Baker T. C. Ochieng' S. A. Cossé A. A. Lee S. G. Todd J. L. Quero C.. (2004). A comparison of responses from olfactory receptor neurons of Heliothis subflexa and *Heliothis virescens* to components of their sex pheromone. J. Comp. Physiol. A Neuroethol. Sens. Neural Behav. Physiol. 190, 155–165. 10.1007/s00359-003-0483-2 14689220

[B7] Bergström G. Lundgren L. (1973). Androconial secretion of three species of butterflies of the genus *Pieris* (Lep., Pieridae). Zoon 1, 67–75.

[B8] Birch M. C. Poppy G. M. Baker T. C. (1990). Scents and eversible scent structures of male moths. Annu. Rev. Entomol. 35, 25–58. 10.1146/annurev.en.35.010190.000325

[B9] Carde R. T. Roelofs W. L. Harrison R. G. Vawter A. T. Brussard P. F. Mutuura A.. (1978). European corn borer: pheromone polymorphism or sibling species? Science 199, 555–556. 10.1126/science.199.4328.555 17750022

[B10] Carlsson M. A. Bisch-Knaden S. Schäpers A. Mozuraitis R. Hansson B. S. Janz N. (2011). Odour maps in the brain of butterflies with divergent host-plant preferences. PLoS ONE 6:e24025. 10.1371/journal.pone.0024025 21901154 PMC3162027

[B11] Carlsson M. A. Chong K. Y. Daniels W. Hansson B. S. Pearce T. C. (2007). Component information is preserved in glomerular responses to binary odor mixtures in the moth *Spodoptera littoralis*. Chem. Senses 32, 433–443. 10.1093/chemse/bjm009 17400588

[B12] Carlsson M. A. Hansson B. S. (2002). Responses in highly selective sensory neurons to blends of pheromone components in the moth *Agrotis segetum*. J. Insect Physiol. 48, 443–451. 10.1016/S0022-1910(02)00065-3 12770093

[B13] Carlsson M. A. Hansson B. S. (2003). Dose-response characteristics of glomerular activity in the moth antennal lobe. Chem. Senses 28, 269–278. 10.1093/chemse/28.4.269 12771013

[B14] Carlsson M. A. Knüsel P. Verschure P. F. Hansson B. S. (2005). Spatio-temporal Ca^2+^ dynamics of moth olfactory projection neurones. Eur. J. Neurosci. 22, 647–657. 10.1111/j.1460-9568.2005.04239.x 16101746

[B15] Couto A. Alenius M. Dickson B. J. (2005). Molecular, anatomical, and functional organization of the *Drosophila* olfactory system. Curr. Biol. 15, 1535–1547. 10.1016/j.cub.2005.07.034 16139208

[B16] Datta S. R. Vasconcelos M. L. Ruta V. Luo S. Wong A. Demir E. (2008). The *Drosophila* pheromone cVA activates a sexually dimorphic neural circuit. Nature 452, 473–477. 10.1038/nature06808 18305480

[B17] Deisig N. Giurfa M. Lachnit H. Sandoz J. C. (2006). Neural representation of olfactory mixtures in the honeybee antennal lobe. Eur. J. Neurosci. 24, 1161–1174. 10.1111/j.1460-9568.2006.04959.x 16930442

[B18] Deisig N. Giurfa M. Sandoz J. C. (2010). Antennal lobe processing increases separability of odor mixture representations in the honeybee. J. Neurophysiol. 103, 2185–2194. 10.1152/jn.00342.2009 20181736

[B19] Fishilevich E. Vosshall L. B. (2005). Genetic and functional subdivision of the *Drosophila* antennal lobe. Curr. Biol. 15, 1548–1553. 10.1016/j.cub.2005.07.066 16139209

[B20] Forsberg J. Wiklund C. (1989). Mating in the Afternoon - Time-Saving in Courtship and Remating by Females of a Polyandrous Butterfly *Pieris napi* L. Behav. Ecol. Sociobiol. 25, 349–356. 10.1007/BF00302992

[B21] Galizia C. G. Nägler K. Hölldobler B. Menzel R. (1998). Odour coding is bilaterally symmetrical in the antennal lobes of honeybees (*Apis mellifera*). Eur. J. Neurosci. 10, 2964–2974. 10.1111/j.1460-9568.1998.00303.x 9758166

[B22] Galizia C. G. Sachse S. Rappert A. Menzel R. (1999). The glomerular code for odor representation is species specific in the honeybee *Apis mellifera*. Nat. Neurosci. 2, 473–478. 10.1038/8144 10321253

[B23] Hansson B. S. Christensen T. A. (1999). Functional characteristics of the antennal lobe, in Insect Olfaction, ed Hansson B. S. (Berlin: Springer), 125–161.

[B24] Hansson B. S. Ljungberg H. Hallberg E. Löfstedt C. (1992). Functional specialization of olfactory glomeruli in a moth. Science 256, 1313–1315. 10.1126/science.1598574 1598574

[B25] Hillier N. K. Vickers N. J. (2011). Hairpencil volatiles influence inter-specific courtship and mating between two related moth species. J. Chem. Ecol. 37, 1127–1136. 10.1007/s10886-011-0017-2 21948202

[B26] Klun J. A. Plimmer J. R. Bierl-Leonhardt B. A. Sparks A. N. Chapman O. L. (1979). Trace chemicals - essence of sexual communication-systems in *Heliothis species*. Science 204, 1328–1330. 10.1126/science.204.4399.1328 17813171

[B27] Klun J. A. Plimmer J. R. Bierlleonhardt B. A. Sparks A. N. Primiani M. Chapman O. L. (1980). Sex-pheromone chemistry of female corn-earworm moth, *Heliothis zea*. J. Chem. Ecol. 6, 165–175. 10.1007/BF00987535

[B28] Larsdotter-Mellström H. Eriksson K. Janz N. Nylin S. Carlsson M. A. (2016). Male butterflies use an anti-aphrodisiac pheromone to tailor ejaculates. Funct. Ecol. 30, 255–261. 10.1111/1365-2435.12521

[B29] Larsdotter-Mellström H. Murtazina R. Borg-Karlson A. K. Wiklund C. (2012). Timing of male sex pheromones biosynthesis in a butterfly - different dynamics under direct or diapaused development. J. Chem. Ecol. 38, 584–591. 10.1007/s10886-012-0126-6 22555771

[B30] Larsdotter-Mellström H. Wiklund C. (2009). Males use sex pheromone assessment to tailor ejaculates to risk of sperm competition in a butterfly. Behav. Ecol. 20, 1147–1151. 10.1093/beheco/arp109

[B31] Lee S. G. Carlsson M. A. Hansson B. S. Todd J. L. Baker T. C. (2006). Antennal lobe projection destinations of *Helicoverpa zea* male olfactory receptor neurons responsive to heliothine sex pheromone components. J. Comp. Physiol. A Neuroethol. Sens. Neural Behav. Physiol. 192, 351–363. 10.1007/s00359-005-0071-8 16308703

[B32] Lei H. Hansson B. S. (1999). Central processing of pulsed pheromone signals by antennal lobe neurons in the male moth *Agrotis segetum*. J. Neurophysiol. 81, 1113–1122. 10085338 10.1152/jn.1999.81.3.1113

[B33] Liu Y. Liu C. Lin K. Wang G. (2013). Functional specificity of sex pheromone receptors in the cotton bollworm *Helicoverpa armigera*. PLoS ONE 8:e62094 10.1371/journal.pone.0062094 23614018 PMC3626661

[B34] Martin J. P. Lei H. Riffell J. A. Hildebrand J. G. (2013). Synchronous firing of antennal-lobe projection neurons encodes the behaviorally effective ratio of sex-pheromone components in male *Manduca sexta*. J. Comp. Physiol. A Neuroethol. Sens. Neural. Behav. Physiol. 199, 963–979. 10.1007/s00359-013-0849-z 24002682 PMC3840155

[B35] Martins P. Sbaite P. Benites C. Maciel M. (2011). Thermal characterization of orange, lemongrass, and basil essential oils, in International Conference on Chemical and Process Engineering, Vol. 24 (Florence), 463–468.

[B36] Riffell J. A. Lei H. Hildebrand J. G. (2009). Neural correlates of behavior in the moth *Manduca sexta* in response to complex odors. Proc. Natl. Acad. Sci. U.S.A. 106, 19219–19226. 10.1073/pnas.0910592106 19907000 PMC2780752

[B37] Roelofs W. Glover T. Tang X. H. Sreng I. Robbins P. Eckenrode C. (1987). Sex pheromone production and perception in European corn borer moths is determined by both autosomal and sex-linked genes. Proc. Natl. Acad. Sci. U.S.A. 84, 7585–7589. 10.1073/pnas.84.21.7585 16593886 PMC299344

[B38] Roelofs W. L. Liu W. T. Hao G. X. Jiao H. M. Rooney A. P. Linn C. E. Jr. (2002). Evolution of moth sex pheromones via ancestral genes. Proc. Natl. Acad. Sci. U.S.A. 99, 13621–13626. 10.1073/pnas.152445399 12237399 PMC129724

[B39] Rouyar A. Deisig N. Dupuy F. Limousin D. Wycke M. A. Renou M. (2015). Unexpected plant odor responses in a moth pheromone system. Front. Physiol. 6:148 10.3389/fphys.2015.00148 PMC4429231 26029117

[B40] Sachse S. Galizia C. G. (2003). The coding of odour-intensity in the honeybee antennal lobe: local computation optimizes odour representation. Eur. J. Neurosci. 18, 2119–2132. 10.1046/j.1460-9568.2003.02931.x 14622173

[B41] Sarto i Monteys V. Acín P. Rosell G. Quero C. Jiménez M. A. Guerrero A. (2012). Moths behaving like butterflies: evolutionary loss of long range attractant pheromones in castniid moths: a *Paysandisia archon* model. PLoS ONE 7:e29282 10.1371/journal.pone.0029282 22238600 PMC3251578

[B42] Silbering A. F. Galizia C. G. (2007). Processing of odor mixtures in the *Drosophila* antennal lobe reveals both global inhibition and glomerulus-specific interactions. J. Neurosci. 27, 11966–11977. 10.1523/JNEUROSCI.3099-07.2007 17978037 PMC6673347

[B43] Sparks A. N. Carpenter J. E. Klun J. A. Mullinix B. G. (1979). Field responses of male *Heliothis Zea* (Boddie) (Lepidoptera, Noctuidae) to pheromonal stimuli and trap design. J. Ga. Entomol. Soc. 14, 318–325.

[B44] Stelinski L. Holdcraft R. Rodriguez-Saona C. (2014). Female moth calling and flight behavior are altered hours following pheromone autodetection: possible implications for practical management with mating disruption. Insects 5, 459–473. 10.3390/insects5020459 26462694 PMC4592591

[B45] Wyatt T. D. (2014). Pheromones and Animal Behavior: Chemical Signals and Signatures, 2nd Edn. Cambridge: Cambridge University Press.

[B46] Yildizhan S. van Loon J. Sramkova A. Ayasse M. Arsene C. ten Broeke C. (2009). Aphrodisiac pheromones from the wings of the small cabbage white and large cabbage white butterflies, *Pieris rapae* and *Pieris brassicae*. Chembiochem 10, 1666–1677. 10.1002/cbic.200900183 19533715

